# Myopericarditis and leishmaniasis: a case report

**DOI:** 10.1093/ehjcr/ytag315

**Published:** 2026-05-05

**Authors:** Maria B Cantos, Diego X Chango, Juan Bravo, Sebastian Garcia-Zamora, Adrian Baranchuk

**Affiliations:** Department of Cardiology, Santa Ana Clinic, Cuenca 010107, Ecuador; Department of Cardiology, Santa Ana Clinic, Cuenca 010107, Ecuador; Department of Cardiology, Mount Sinai Hospital, Cuenca 010107, Ecuador; Department of Cardiology, Mount Sinai Hospital, Cuenca 010107, Ecuador; Department of Cardiology, Delta Clinic, Rosario B2810, Argentina; Department of Cardiology, Kingston University, Ontario K7K1A7, Canada

**Keywords:** Leishmaniasis, Myocarditis, Pericarditis, Cardiac magnetic resonance, Case report

## Abstract

**Background:**

Leishmaniasis belongs to a group of neglected tropical diseases worldwide, causing significant morbidity and mortality. Cardiovascular complications are rare; however, when they occur, they can be severe. Pericardial involvement with varying degrees of effusion is one of its main complications, whereas myocardial involvement remains poorly documented and not well established.

**Case summary:**

In our case report, we present a young woman from the Amazon region, diagnosed with cutaneous leishmaniasis and exertional dyspnoea. Cardiac magnetic resonance imaging revealed active pericarditis with significant pericardial effusion and myocardial involvement characterized by non-ischaemic late gadolinium enhancement. The patient remains under clinical surveillance, improving pericardial effusion with specific treatment.

**Discussion:**

Our case highlights a possible association between leishmaniasis and myopericardial involvement and underscores the importance of considering this diagnosis in patients from endemic regions, while acknowledging that a causal relationship cannot be established.

Learning points

- Cardiac involvement in leishmaniasis is uncommon but may result in significant morbidity and mortality.
- Cardiac magnetic resonance imaging is a valuable tool for comprehensive evaluation, particularly in cases of pericarditis or myocarditis.
- Regular cardiac imaging is essential for monitoring disease progression and assessing treatment response in patients with cardiac Leishmaniasis.

## Introduction

Leishmaniasis is a neglected tropical disease caused by protozoa of the genus *Leishmania*, transmitted primarily through the bite of infected sandflies and disproportionately affecting socioeconomically vulnerable populations. Clinical manifestations are heterogeneous, ranging from cutaneous to visceral forms, the latter being the most severe. Although the parasite may involve multiple organs, cardiac involvement is rare and is thought to be mediated by infection-related inflammatory processes, most commonly presenting as pericardial disease. Early recognition of cardiac involvement may be clinically relevant to prevent potentially serious complications. Cardiovascular magnetic resonance (CMR) is a valuable imaging modality for comprehensive assessment of cardiac anatomy, function, and tissue characterization, enabling accurate detection of myocardial and pericardial inflammation. Its role is particularly relevant in uncommon or under-recognized causes of inflammatory heart disease, where conventional diagnostic tools may be insufficient.

## Summary figure

**Figure:**
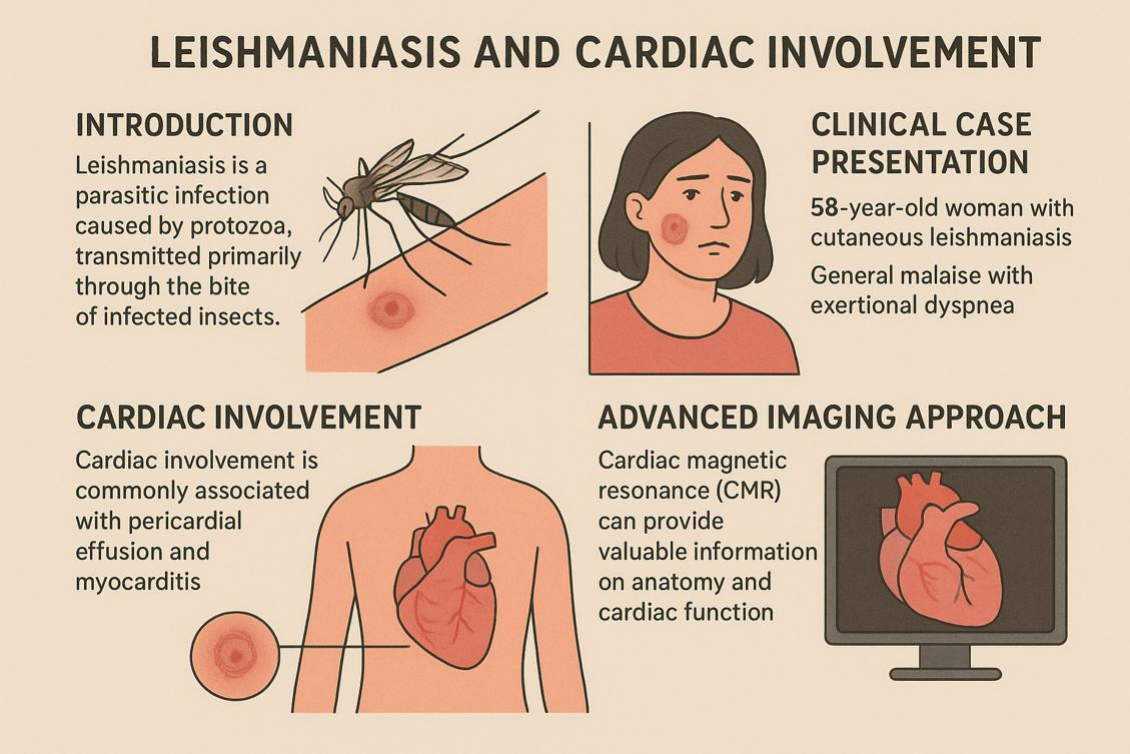


## History of presentation

**Figure 1:**
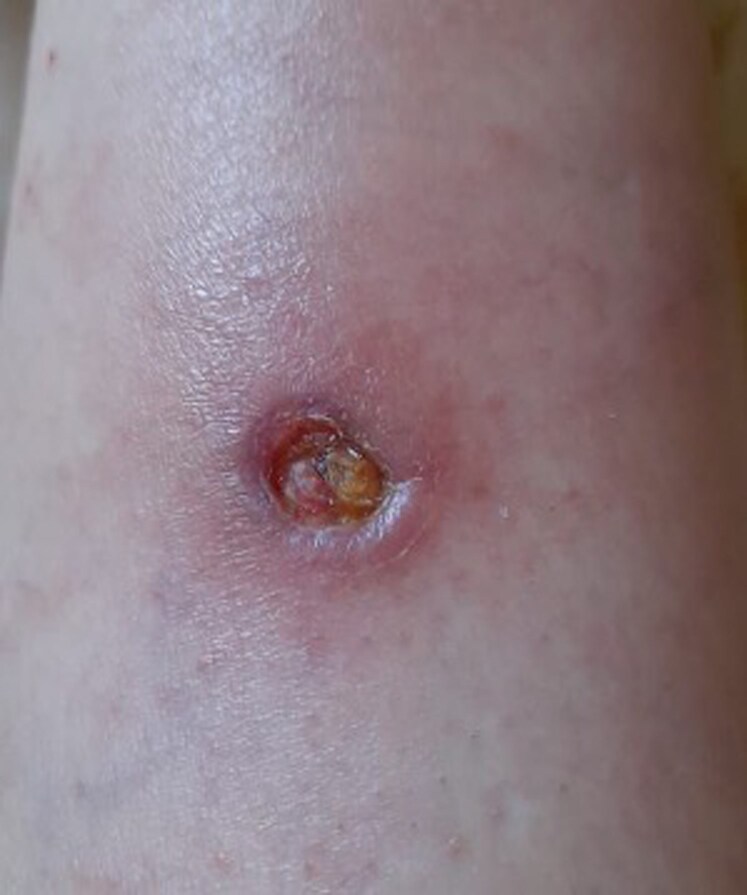
Cutaneous leishmaniasis with skin lesion showing an ulceration with raised edges and a central crater. The ulcer was covered with a crust.

We present the case of a 58-year-old woman residing in the Amazon region who presented with clinical features consistent with cutaneous leishmaniasis, including ulcerated skin lesions of approximately 3 months’ duration (*Figure 1*). The patient also reported exertional dyspnoea corresponding to New York Heart Association (NYHA) functional Class II, associated with general malaise, which prompted referral for cardiovascular evaluation. On physical examination, heart sounds were normal without murmurs, and there were no signs of congestion or other clinically relevant findings.

## Past medical history

The patient had no significant past medical or surgical history.

## Investigations

**Figure 2:**
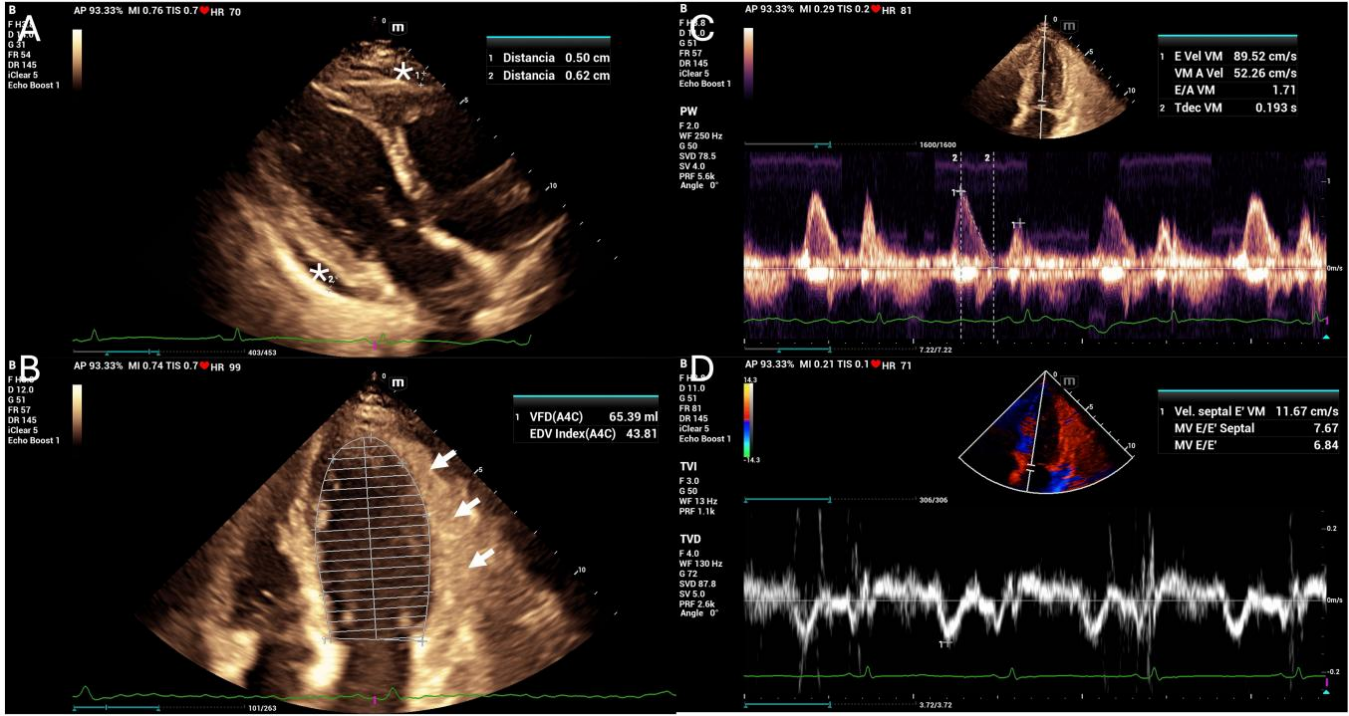
Transthoracic echocardiogram. *(A)* Right parasternal long-axis image showing pericardial effusion (asterisks) and normal-sized left cavities. *(B)* Apical four-chamber image showing pericardial effusion with areas of pericardial hyper-refringence (white arrows). *(C and D)* Pulsed and tissue Doppler showing a preserved E/A and septal ratio without flow variability.

**Figure 3:**
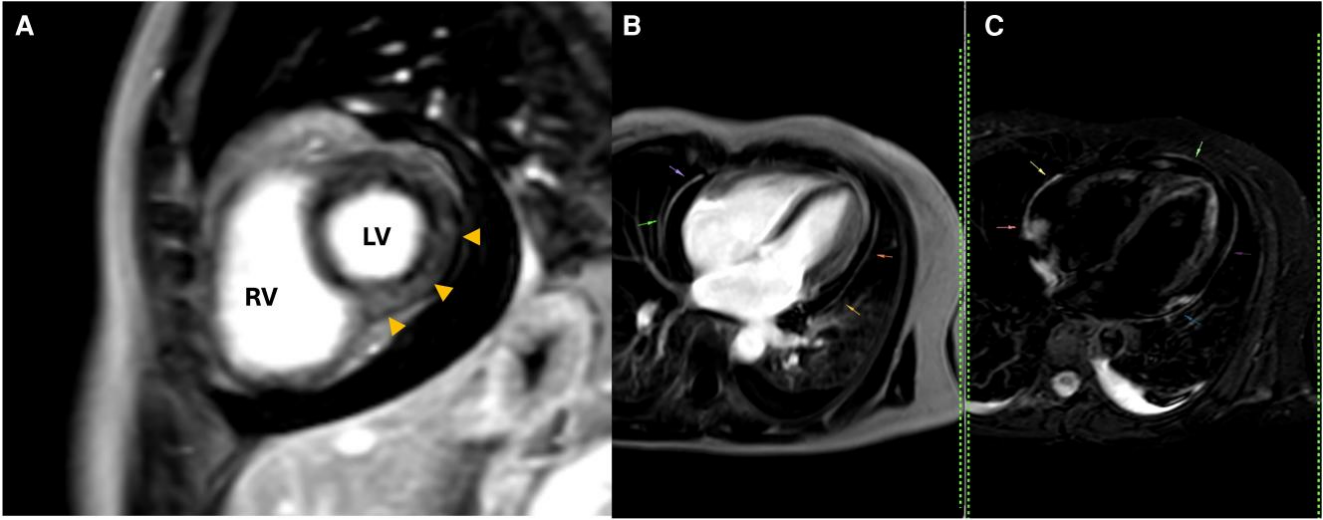
Cardiovascular magnetic resonance images demonstrating. *(A)* Subepicardial non-ischaemic late gadolinium enhancement in the inferior and inferolateral basal and mid left ventricular segments (arrows), consistent with myocardial inflammatory involvement. *(B and C)* Four-chamber view image in late post-contrast T1-weighted and T2-weighted showing late gadolinium enhancement oedema at the level of the pericardium.

The diagnosis of cutaneous leishmaniasis was confirmed by direct smear microscopy, which demonstrated the presence of intracellular amastigotes. A 12-lead electrocardiogram demonstrated sinus rhythm, without conduction abnormalities or other significant pathological findings. Laboratory evaluation revealed elevated cardiac biomarkers, with a troponin level of 45 ng/L (reference <14 ng/L) and an NT-proBNP level of 653 pg/ml (reference <125 pg/ml). Transthoracic echocardiography revealed a moderate circumferential pericardial effusion, predominantly located along the posterior and lateral pericardial spaces, with echogenic fibrin strands. Biventricular systolic function was preserved, diastolic function was normal, and colour Doppler imaging showed no evidence of haemodynamic compromise or features of cardiac tamponade (*Figure 2*; Supplementary material online, *Videos S1*–*S3*). Further evaluation with CMR demonstrated normal chamber volumes and preserved biventricular systolic function, with a left ventricular ejection fraction of 69% and a right ventricular ejection fraction of 66%. Indexed left ventricular end-diastolic and end-systolic volumes were 54 and 16 ml/m^2^, respectively, and indexed right ventricular end-diastolic and end-systolic volumes were 52 and 18 ml/m^2^. Significant findings included a moderate circumferential pericardial effusion without haemodynamic compromise (see Supplementary material online, *Videos S4*–*S6*). Pericardial thickness was normal, with evidence of focal oedema on T2-weighted sequences and late gadolinium enhancement on post-contrast T1-weighted images, consistent with active pericarditis and associated myocardial involvement (*Figure 3*). The Lake Louise criteria demonstrated features consistent with active myocarditis, including myocardial oedema and hyperaemia on T1- and T2-based sequences. Non-ischaemic late gadolinium enhancement with a subepicardial distribution was observed in the inferior and inferolateral segments of the basal and mid left ventricular walls, suggestive of myocardial inflammatory involvement in the clinical context of systemic infection (*Figure 3*).

## Management

Treatment with intramuscular meglumine antimoniate (75 mg/kg/day), administered every 8 h, was initiated for a total duration of 20 days. Concomitant cardiac therapy, including anti-inflammatory drugs and colchicine, was initiated alongside treatment of the underlying infectious disease.

## Outcome and follow-up

**Figure 4:**
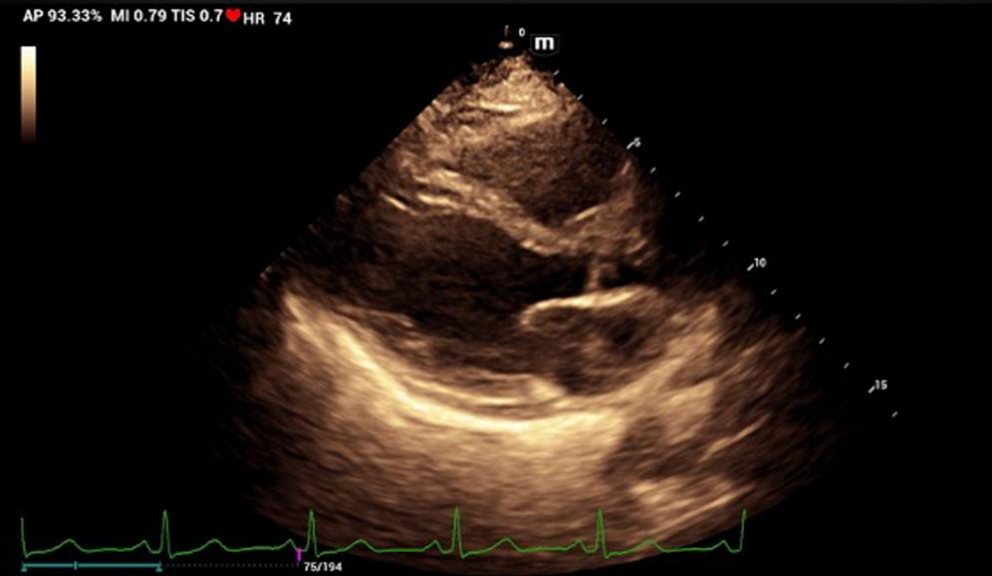
Echocardiographic evaluation showed no pericardial effusion.

The patient remained clinically stable during follow-up, with no worsening of symptoms or elevation of cardiac biomarkers. Follow-up transthoracic echocardiography demonstrated complete resolution of the pericardial effusion, and the patient reported significant symptomatic improvement (*Figure 4*; Supplementary material online, *Videos S7* and *S8*).

## Discussion

Leishmaniasis is a neglected tropical disease with a broad spectrum of clinical manifestations, while cardiac involvement remains distinctly uncommon. When present, it is thought to be mediated by infection-related inflammatory mechanisms and most frequently manifests as pericardial disease, particularly pericardial effusion.^1–3^ Myocardial involvement is far less well characterized and has been reported mainly in isolated case reports or in immunocompromised patients, especially those co-infected with HIV.^3–5^ In this case, cardiovascular magnetic resonance played a central diagnostic role by confirming active pericarditis and demonstrating concomitant myocardial involvement through non-ischaemic subepicardial late gadolinium enhancement. This comprehensive tissue characterization supported the diagnosis of myopericarditis, which could not have been reliably established using electrocardiography or echocardiography alone.^6–9^ Although the Lake Louise criteria are primarily validated for viral myocarditis, the combination of myocardial oedema and characteristic late gadolinium enhancement strongly supported inflammatory myocardial involvement in this clinical context. The principal novelty of this report lies in the documentation of CMR-confirmed myopericarditis in the setting of leishmaniasis in an immunocompetent patient. This finding expands the current understanding of cardiac manifestations associated with leishmaniasis and underscores the importance of considering myocardial involvement even in the absence of immunosuppression. From a clinical perspective, this case highlights the value of advanced imaging in enabling early and accurate diagnosis, guiding appropriate therapy, and potentially preventing adverse cardiovascular outcomes in patients with parasitic infections.

## Conclusion

This case highlights a possible association between leishmaniasis and myocardial involvement, although a direct causal relationship cannot be established. Cardiovascular magnetic resonance plays a key role in identifying myocardial and pericardial inflammation, particularly in atypical or under-recognized infectious contexts. Further studies are needed to better understand the potential cardiac involvement in leishmaniasis.

## Supplementary Material

ytag315_Supplementary_Data

## Data Availability

All data supporting the findings of this study are included within the article. Further details are available from the corresponding author upon reasonable request.

## Lead author biography

Maria B. Moscoso is a medical staff of Santa Ana Clinic, Cuenca, Ecuador.

## Notes

**Consent:** Written informed consent was obtained from the patient for the publication of this case report and any accompanying images, in accordance with COPE guidelines.
